# On the predictive utility of animal models of osteoarthritis

**DOI:** 10.1186/s13075-015-0747-6

**Published:** 2015-09-14

**Authors:** Anne-Marie Malfait, Christopher B. Little

**Affiliations:** Department of Medicine, Division of Rheumatology, and Department of Biochemistry, Rush University Medical Center, Chicago, IL 60612 USA; Raymond Purves Bone and Joint Research Laboratories, Kolling Institute of Medical Research, Institute of Bone and Joint Research, University of Sydney at Royal North Shore Hospital, St Leonards, NSW 2065 Australia

## Abstract

Animal models of osteoarthritis are extensively used for investigating disease pathways and for preclinical testing of novel therapies. Their predictive utility, however, has often been questioned, mainly because preclinical efficacy of novel therapeutics is poorly translated in clinical trials. In the current narrative review, we consider the preclinical models that were used to support undertaking clinical trials for disease-modifying osteoarthritis drugs, and compare outcomes between clinical and preclinical studies. We discuss this in light of the 1999 Food and Drug Administration draft guidelines for industry for use in the development of drugs, devices, and biological products intended for the treatment of osteoarthritis, which raised five considerations on the usefulness of osteoarthritis models. We systematically discuss what has been learnt regarding these five points since 1999, with emphasis on replicating distinct risk factors and subtypes of human osteoarthritis, and on comprehensive evaluation of the disease in animals, including pathology of all joint tissues, biomarker analysis, and assessment of pain and joint function. Finally, we discuss lessons learnt and propose some recommendations for how the evidence from preclinical research might be strengthened with a view to improving success in clinical translation.

## Introduction

The current practice of translational biomedical research is failing its end-users, with as much as 90 % of ‘highly promising basic science discoveries failing to enter routine clinical use within 20 years’ [1]. As a result, it has been estimated that 85 % of research resources are ‘wasted’ [2]. That the majority of basic and preclinical medical research does not move to clinical trials, let alone lead to substantive improvements in patient health, challenges the relevance of the present translational discovery and development model [3, 4]. In an attempt to rationalize selection of promising therapeutic targets, metrics to evaluate ‘translatability’ have been developed [5, 6] and retrospectively validated [7]. Interestingly, in this proposed scoring system, ‘starting evidence’, which includes data from *in vitro* research, genetic modification in mice, and preclinical animal models of disease, only contributes a maximum of up to 11 % of the total translatability score. Despite this, a detailed analysis by one large pharmaceutical company of failures in their drug development pipeline showed that the most common reason (40 % versus 29 % for next most common) was inadequate linkage of the molecular/cellular target with the disease and no validated models [8]. Thus, while other metrics such as human genetic data, availability of biomarkers, and early phase clinical trial outcomes are clearly critical for successful translation, drug development programs will fail if their biological basis (that is, ‘the starting evidence’) is not sound. A cornerstone of strong scientific foundation for therapeutic development in all areas of medicine is the use of valid preclinical animal models of human disease, with predictive utility for research into disease pathways as well as for drug testing.

**Fig. 1:**
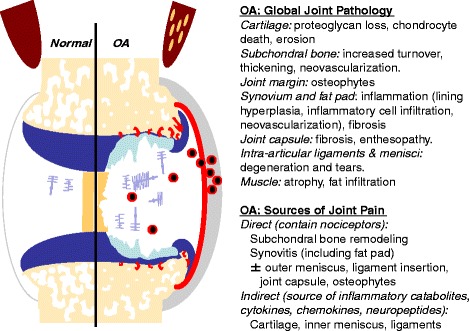
Osteoarthritis (OA) is a disease of the whole joint with pathology in all articular tissues and associated skeletal muscle. Pain in the OA joint can only arise directly from innervated tissues, but sensory neurons may be activated by factors released from aneural joint tissues

In 1999, the US Food and Drug Administration (FDA) provided draft guidelines for industry for use in the development of drugs, devices, and biological products intended for the treatment of osteoarthritis (OA) [9]. This nine-page document contains a paragraph on the use of preclinical models in OA, which surprisingly is lacking in the equivalent European guidelines [10]. The brief section (excerpt in Box 1) in the FDA document raises a number of specific issues that speak largely to how well any proposed animal model of OA mimics the human disease, and will therefore be predictive of therapeutic outcome in clinical trials and ultimately medical practice. Since the publication of the FDA document, 15 years ago with no further updates, OA research has witnessed many advances, both basic and clinical. For one, the concept of OA has broadened from a cartilage-driven disease to ‘a whole joint disease’ (Fig. 1). Magnetic resonance imaging (MRI) of painful OA knees clearly shows involvement of all joint tissues [11] and detailed molecular studies reveal that different joint tissues may all contribute to pathogenesis [12]. Consequently, many new targets that act on distinct aspects of OA pathology have been identified, leading to several clinical trials of putative disease-modifying OA drugs (DMOADs) that target various pathways in different joint tissues (Table 1). In general, outcomes of these trials have been disappointing, and there are still no FDA-approved DMOADs on the market. One question that arises is whether we could have predicted trial outcomes better using more appropriate preclinical models? Therefore, in the current narrative review, we systematically discuss the five considerations raised by the FDA regarding preclinical models of OA (Box 1), and review what, if anything, has been learnt since 1999. We consider the preclinical models that were used to support undertaking clinical trials for DMOADs, and compare outcomes between clinical and preclinical studies. Finally, we discuss lessons learnt and propose some recommendations for how the evidence from preclinical OA research might be strengthened with a view to improving success in this area of translational medicine.

**Table 1:** Clinical DMOAD trials in knee osteoarthritis (placebo-controlled, peer-reviewed and published since 1999)

Trial	Target	Disease modification	Symptomatic outcome (secondary endpoint)	Preclinical validation in OA model	Structural outcome	Symptomatic outcome
Oral salmon calcitonin (n = 1,176 and n = 1,030; 2 years) [145]	SCB	JSW: no effect. No statistically significant effect on biochemical markers of bone (CTX-I) and cartilage degradation (CTX-II)	WOMAC: no statistically significant effect	Rat MNX and MNX/OVX [146]: 8 weeks, treatment at start	Joint protection, serum CTX-II ↓	NA
				Dog ACLT: Rx at surgery, 84 days (nasal delivery) [147]	Joint protection (no effect on osteophytes)	NA
				DMM in mice overexpressing salmon calcitonin [148]	OARSI score ↓ 7 weeks after DMM	NA
Intra-articular rFGF18 (n = 168; 12 months) [149]	Cartilage (anabolic)	Primary endpoint, (reduction of cartilage loss in the central medial femorotibial compartment on MRI) not met. Secondary structural endpoints were met	WOMAC: improved	Rat MMT: 6 weeks; Rx start week 3 [150]	Increased thickness of the articular surface of the medial tibial plateau. Reduced degeneration scores	NA
Strontium ranelate (n = 1,371; 3 years) [151]	SCB	JSW: fewer radiographic progressors (both low and high dose)	Beneficial effects on symptoms (high dose only)	Dog ACLT [152]: 16 weeks; Rx start week 4	Cartilage lesions ↓ (macrosc/histol), SCB thickening ↓ (histomorphometry), serum CTXII ↓	NA
				Rat MMT [153]: 3 or 6 weeks; Rx start at surgery	Cartilage degeneration ↓, SCB remodeling ↓	NA
SD6010, oral selective iNOS inhibitor (n = 1,048; 2 years) [154]	Cartilage	JSW: no effect	No effect on pain or function	Dog ACLT [155, 156]: 10 or 12 weeks; Rx start at surgery	Cartilage lesions ↓ (macrosc/histol), osteophytes ↓, synovial inflammation ↓	NA
				Collagenase induced arthritis in *Nos2* null mice [157]	Cartilage proteoglycan loss ↓, cartilage lesions ↓, osteophytes ↓	NA
				Rat MMT model [158]	NA	Reversal of mechanical allodynia and reversal of WBD 3 hours after drug administration
Zoledronic acid (n = 59; single infusion; 6 and12 month follow-up) [77]	SCB	MRI BML area: reduction in total BML area significant at 6 but not 12 months	VAS pain scores ↓ at 6 months, but not KOOS	Rat MIA [92], rat MMT [70]		
				a) prophylactic	Joint preservation	Reversal of WBD
				b) therapeutic (early or late intervention)	Partial preservation, diminishes with late intervention	Partial effect, diminishing with late intervention
Vitamin D3 (n = 146; 2 years) [159]	SCB cartilage	MRI cartilage volume: no effect	WOMAC: no effect	Rat pMNX [160]: prophylactic; 40 days	Inconclusive	NA
				Osteochondrosis/OA in pigs; Vit D3 in diet [161]	No effect on OA incidence or severity of OA lesions, or cartilage biochemistry	NA
Licofelone (5-LOX and COX inhibitor; n = 355; 2 years; not placebo controlled but compared with NSAID) [162]	Inflammation	JSW: no effect	WOMAC: pain improved	Dog ACLT [163]: 12 weeks; Rx start week 4	MRI cartilage volume ↑, cartilage damage and osteophytes ↓(macroscopic evaluation only)	NA
Risedronate	SCB			DH guinea pig [164]: up to 24 weeks	OARSI score: no effect. Serum CTX-II ↓	NA
				NZW rabbits ACLT [165]: 11 weeks; Rx start week 3	Loss of cartilage ↓, SCB damage ↓, serum CTX-II ↓	NA
N = 284 (1 year) [166]		JSW: trend toward improvement. Cartilage degradation and bone resorption markers ↓	WOMAC ↓			
N = 2,483 (2 years) [167]		JSW: no effect. uCTXII ↓	WOMAC : no effect			
N =1,232 (2 years) [168]		Preserved SCB integrity	NA			
Broad-spectrum MMP inhibitor (n =401; 1 year) [169]	Cartilage	JSW: no effect	No effect on pain	rat MIA [170]: 3 weeks; Rx during first week	Cartilage damage ↓	NA
				STR/Ort mice [171]: 12 weeks	Improved radiographic score and less cartilage and bone damage	NA
Doxycycline (n = 403; 30 months) [172]	Cartilage	JSW: slowed JSN in ipsilateral knee	No effect on pain	Dog ACLT (after dorsal root ganglionectomy) [173]: 8 weeks; Rx at start	Less damage on femoral condyle. No effect on tibial plateau or osteophytes	NA
				DH guinea pig [174]: 9 months old; 66 days	Less cartilage volume loss (MRI)	NA
				DMM (mouse) [175]: 4 weeks; Rx at start	Less cartilage loss	NA

ACLT, anterior cruciate ligament transection; BML, bone marrow lesion; COX, cyclo-oxygenase; CTX, C-terminal crosslinked telopeptide type II collagen; DH, Dunkin-Hartley; DMM, destabilization of the medial meniscus; DMOAD, disease-modifying osteoarthritis drug; iNOS, inducible nitric oxide synthase; JSN, joint space narrowing; JSW, joint space width; LOX, lipoxygenase; KOOS, knee injury and osteoarthritis outcome score; MIA, mono-iodoacetate; MMP, matrix metalloproteinase; MMT, medial meniscal tear; MNX, meniscectomy; MRI, magnetic resonance imaging; NA, not applicable; NSAID, nonsteroidal anti-inflammatory drug; OA, osteoarthritis; OARSI, Osteoarthritis Research Society International; OVX, ovariectomy; Rx, treatment; SCB, subchondral bone; VAS, visual analog score; WBD, weight bearing deficit; WOMAC, Western Ontario and McMaster Osteoarthritis Index

## How accurately does the model replicate human osteoarthritis?

OA is no longer considered a single disorder; rather, it is a collection of different disease phenotypes that share certain clinical and pathological features [13]. Consequently the responses to treatment, and indeed the appropriate therapeutic targets in these different OA phenotypes, may be quite distinct [14, 15]. Suggested clinically relevant stratification approaches into OA phenotypes that may warrant distinct therapeutic strategies include: (i) cause or mechanism of onset (post-traumatic, age-associated, metabolic, or genetic); (ii) tissues affected (cartilage erosion, bone erosion or formation, synovitis/inflammation, muscle atrophy); (iii) progression (stage and rate); and (iv) symptoms (pain, disability) [16–20]. In light of these recent refinements in OA stratification, the choice of an ‘appropriate’ animal model will strongly depend on which type of human OA one wishes to replicate. Indeed, studies in genetically modified mice have revealed that in approximately one-third of cases the therapeutic outcome of an identical ‘intervention’ was dissimilar in experimental models mimicking different OA subtypes [21, 22]. This difference in outcome varied from an intervention being effective in one subtype but not another (for example, ablation of S100A8/9 inhibits cartilage erosion and osteophyte development only in experimental OA with a significant inflammatory component [23, 24]) to actually having opposite effects (for example, IL-6 ablation is protective in post-traumatic OA in young mice [25] but worsens age-associated OA [26]).

Age is one of the strongest risk factors for OA. Ageing causes changes in chondrocytes and articular cartilage, as well as in other joint tissues (meniscus, ligaments, bone, and synovium) and these age-related changes contribute to OA pathogenesis (reviewed in [27]). Radiographic changes in the joint become more common with age and OA often becomes symptomatic [27]. In contrast, most preclinical studies are conducted in young animals. For example, in the most widely used murine model of OA, the destabilization of the medial meniscus (DMM) model [28], the surgery is usually performed on 10- to 12-week-old mice. However, comparison of 12-week-old and 12-month-old mice revealed that age affects the basal pattern of gene expression in joint tissues [29], and when DMM is performed on 12-month-old animals, the ensuing OA is more severe than in young mice [29]. Rodents also develop spontaneous OA with age [30]. Thus, when modeling molecular mechanisms to define therapeutic targets in mice and rats, animal age should be a critical consideration. Response to specific compounds, such as putative DMOADs or novel OA analgesics, has not yet been directly compared in young versus old animals using the same OA model. There has been some comparison of the effect of a given genetic modification on induced post-traumatic versus spontaneous age-associated OA (reviewed in [21]) but there have been no studies that directly compare the effect of a specific gene on post-traumatic OA (induced by DMM, for example) in young versus old mice. Such studies would likely enhance our appreciation of the predictive potential of specific models with respect to different OA phenotypes.

While age remains perhaps the strongest predictor of OA development, overweight and obesity not only significantly increase the risk of incident hip and knee OA, particularly in women, but also its radiographic progression (reviewed in [31]). Studies in experimental animals have similarly shown increased OA incidence and severity with obesity, and it is apparent that this is not simply driven by increased mechanical loading of joints [32]. High-fat diets in the absence of obesity, and elevation of specific lipid components increase OA to a similar extent in laboratory animals, highlighting the complexity of the metabolic syndrome and the role of local and systemic inflammation and specific cytokines, chemokines and adipokines [33–36]. In addition to being a primary risk factor, obesity/metabolic syndrome also exacerbates post-traumatic OA in mice [37, 38], and the OA that occurs spontaneously in *Trpv4*^−/−^ mice [39]. To our knowledge, no studies have evaluated whether high-fat diet/obesity offsets the protective effect of other genetic modifications in mice such as ablation of *Adamts5* or *Mmp13*. As suggested above with ageing, such studies would be of value in improving knowledge of the interaction of clinically important OA risk factors and improve the translational utility of data from preclinical models.

Not only does diet-induced obesity increase OA joint pathology in a mouse model, but it also induces anxiety and hyperalgesia, and decreases muscle function and locomotor activity [40]. These global effects are typical of the metabolic OA syndrome in patients, supporting the relevance of this preclinical model to the human condition [41]. In the few reported pharmacological interventions in high-fat diet-induced OA, a statin and a peroxisome proliferator-activated receptor γ agonist reduced pathology in C-reactive protein-transgenic mice [34] and a statin but not another cholesterol lowering agent reduced OA in the APOE*3 L.CETP transgenic hyperlipidemia mouse model [36]. The effect of statins on spontaneous OA in the STR/Ort mouse, which also displays obesity, have been inconsistent [42, 43]. While these divergent effects suggest caution in extrapolation of outcomes to humans, they actually reflect the variability seen in patients. Thus, a population-based study suggested there may be a disease-modifying effect of statins in knee but not hip OA [44]. However, a recent report [45] could not demonstrate association between nodal OA, hip OA or knee OA and use of statins after adjusting for confounders, although use of statins was associated with a lower prevalence of the generalized OA phenotype. In addition to effects of pharmacological agents, moderate exercise reduced OA severity in obese mice, and interestingly this was not associated with altered weight or body fat [46]. These results confirm that increased joint loading is not the primary driver of obesity-associated OA, consistent with outcomes of weight-loss and exercise programs in patients [47]. Weight loss relieves pain in obese OA patients, with a weight loss of at least 10 % providing significant pain reduction [48]. A recent study in individuals with symptomatic knee OA suggested a dose–response relationship between changes in body weight and corresponding changes in pain and physical function [49]. To date, no small animal models of obesity-induced OA have evaluated the effect of specific interventions on pain, but trials in overweight dogs confirm that exercise and weight loss may have a positive effect on pain and gait [50, 51].

Despite sex (in association with age and obesity) being a significant confounding factor for OA risk (reviewed in [52]), this has not been systematically studied and compared in preclinical research, either for pathophysiological or drug studies. Female sex, particularly post-menopausally, is a risk factor for prevalence and severity of OA [53]. As in humans, OA in baboons is more common in males in the younger population, but disease progresses more quickly following menopause so it is more prevalent and severe with age in females [54]. In contrast, in mice [55] and guinea pigs [56], spontaneous/age-associated OA is more common and severe in males than females. This may reflect greater weight gain in older males in these species, and that in animals other than primates, natural menopause and associated changes in bone turnover do not occur, necessitating ovariectomy to mimic the increased OA risk in older female patients (reviewed in [57]). Induced OA in animals may also be more progressive in males such as following DMM in mice [58], and as a result the majority of murine DMM studies are done in males with direct comparison of OA outcome measures or interventions (including genetic modification) in females being uncommon (for an example, see [59]). Increasingly, researchers are testing therapeutic intervention in ovariectomized rodents [60–64]. Pain researchers have long recognized that females are at greater risk for chronic pain, and the International Association for the Study of Pain has recommended the use of female experimental animals [65]. A recent meta-analysis showed that female sex is one of the main risk factors associated with onset of knee pain [66]. Despite the clear importance of sex in OA risk, progression and symptoms in patients, we are not aware of any preclinical studies directly comparing outcomes of therapeutic trials in males versus female animals.

OA can be stratified according to affected tissues (Fig. 1). For instance, a subset of OA patients has high bone turnover and/or low subchondral bone (SCB) density, and as such may be responsive to bone-specific therapies [67]. Variable bone remodeling phenotypes and response to anti-resorptive therapy are also evident in preclinical OA models, depending on: (i) induction method (for example, intra-articular mono-iodoacetate (MIA) induces profound vascular invasion and SCB loss [68, 69]); (ii) stage of disease (early SCB loss followed by formation in a surgical rat model [70]); and (iii) species (for example, OA induced by meniscal injury in mice [61, 71] has a very limited bone resorption phase compared with rats [70, 72]). Like OA patients [17], different preclinical models may show different degrees of inflammation, and this can determine how an experimental treatment affects joint pathology or pain. For example, intra-articularly deposited adipose stem cells protect against cartilage damage and osteophyte size in inflammatory-driven collagenase-induced arthritis but not in the DMM model where synovitis is less pronounced [73]. In a side-by-side comparison in rats [74], meniscectomy resulted in more inflammation than MIA, and this was reflected by a greater analgesic effect of intra-articular triamcinolone, consistent with a greater contribution of synovitis to pain in the surgical model. This sort of side-by-side preclinical study highlights the necessity for selecting the animal model based on the pathological feature of OA one wishes to interrogate.

## What are the structural determinants of pain and loss of function?

In knee OA, population studies support a substantial discordance between radiographic changes and knee pain [75]. Many knee MRI studies, both cross-sectional and longitudinal, have suggested associations of specific structural changes with OA pain, the strongest for synovitis and SCB (Fig. 1) - in particular, MRI-detected bone marrow lesions (reviewed in [76]). Recent clinical trial data suggest that targeting SCB in OA may indeed have an effect on pain (Table 1). A randomized trial with intravenous zoledronic acid, a bisphosphonate used for the treatment of osteoporosis, demonstrated a reduction in the volume of bone marrow lesions, and this was associated with reduced pain in subjects with knee OA [77]. Another osteoporosis drug, strontium ranelate, recently showed disease modification in a large 3-year placebo-controlled trial, and this was associated with a beneficial effect on pain, further supporting the role of bone in OA pathogenesis and symptoms [78, 79]. It has been reported that worsening of MRI-detected synovitis is associated with increased risk of frequent knee pain, but improvement of synovitis is not associated with decreased risk of pain or pain severity [80]. Therapeutic studies have demonstrated significant association between reduced synovitis on MRI and reduced pain in patients with rheumatoid arthritis [81] but not OA [82], suggesting there may be a poorer structure-function relationship in the latter.

To date, the relationship between pain/disability and specific joint structural changes have not been extensively explored across different OA models and species. Yet, targeted pharmacological modulation of specific facets of OA pathology in experimental animals permits dissecting the contribution of different aspects of joint disease to pain. Such studies require measuring pain on the one hand, which can be approached using different assays, including evoked pain responses and spontaneous pain behaviors (reviewed in [83]), and joint pathology on the other hand. In order to determine specific structural correlates to pain, pathology has to be carefully evaluated in all joint tissues, as opposed to a limited evaluation of the cartilage. While cartilage damage remains the primary outcome in most OA models, there has been a clear shift in recent years to include evaluation of other pathological changes in the joint such as osteophytes, SCB remodeling and synovitis [22]. Such comprehensive histopathology can reveal differential effects of a specific gene on joint tissues; for instance, ablation of *Adamts5* or *Mmp13* protects against cartilage damage and SCB sclerosis but not osteophyte formation after DMM [84, 85], while *Ccr5* null mice develop less cartilage degeneration but show no differences in bone or synovial response to surgery [86]. This comprehensive approach to joint pathology is, however, far from the norm across different species and models, and methods for quantifying histopathology in joint tissues other than cartilage have not been standardized [87]. In addition, while muscle weakness and/or wasting is typical in OA patients, potentially playing a role in disease onset and progression and being a target for therapeutic intervention [88, 89], changes in skeletal muscle either as a consequence or cause of OA and associated pain in preclinical models remain largely understudied [90].

The contribution of SCB to OA pain has been examined in some detail in preclinical models of OA treated with different bone-remodeling agents. In rat MIA, pre-emptive alendronate treatment preserved SCB trabecular microarchitecture, decreased bone turnover and had moderate effects on cartilage degradation. These structural effects were accompanied by a positive effect on weight-bearing asymmetry, an indicator of pain [69]. In a canine study where OA was induced by transection of the anterior cruciate ligament, tiludronate treatment from time of surgery for 8 weeks had no effect on the severity of cartilage lesions or osteophytes, but treated dogs had a greater SCB surface and less synovitis than the vehicle-control group. These effects on bone and synovium were accompanied by improved pain behaviors (video-captured) and less gait disability [91]. These studies suggest that SCB bone may be a source of OA pain and targeting the bone may represent a strategy for OA analgesia. Several studies have compared therapeutic efficacy of targeting SCB in early versus later stages of disease, and found that the beneficial effect is greatest in early stages of disease when remodeling is most active. The effects of zoledronate in attenuating bone and cartilage loss and the accompanying weight-bearing asymmetry were more pronounced in prophylactic and early treatment protocols than in a delayed therapeutic setting [92]. Zoledronate had similar effects in the rat medial meniscal tear model [70], but only if administered early in the disease. Interestingly, an independent study recently confirmed the effect of prophylactic treatment with zoledronate in the rat MIA model on joint protection and concomitant partial reversal of weight-bearing deficits, and found this was associated with fewer channels crossing the osteochondral junction [93]. These osteochondral channels, containing blood vessels and small nociceptive neurons, have also been demonstrated in human OA, and may contribute to pain [94]. While pre-emptive administration of the osteoclastogenesis inhibitor osteoprotegerin in rat MIA reduced the number of subchondral osteoclasts, synovitis, and asymmetric weight-bearing, therapeutic treatment prevented further changes in weight-bearing and attenuated osteoclast numbers but not other structural changes in the joint [93].

## Do structural changes correlate with clinical or biochemical markers?

When characterizing a preclinical model, assessing the effect of a particular gene, or evaluating therapeutic efficacy, emphasis has traditionally been on structural assessment of the joint, mostly through macroscopic assessment and histological scoring. The Osteoarthritis Research Society International (OARSI) has published guidelines for histological assessment of OA joints in different species in an attempt to standardize the assessment and reporting of animal studies (see collected papers in *Osteoarthritis and Cartilage* 2010, Supplement 3). Histology, however, does not allow for longitudinal assessment of the joint, and recent years have witnessed tremendous efforts to improve joint imaging in animals. In rodents, microCT, high-field MRI, and an array of contrast, luminescent and fluorescent bio-imaging methods have been developed (for reviews, see [95–97]). Discussion of these methods is beyond the scope of the current review, but preclinical models may play a critical role in identifying imaging markers that are predictive for onset and progression of OA.

One of the great challenges of clinical trials in OA has been the slow progression of disease and a lack of biomarkers that are predictive of OA structural disease onset and progression, and associated pain and disability. Since the writing of the FDA guidelines, emphasis has shifted to diagnosis and treatment of patients with early (preclinical) OA prior to significant/irreversible structural change and/or chronic pain where central sensitization may confound symptom modification [98]. With recent advances in imaging modalities, it has also become apparent that patients may have early osteochondral damage and meniscal tears that are asymptomatic but represent significant risk factors for progressive OA. Thus, the emphasis may shift even further to identifying, and perhaps treating, those that are at higher risk of developing OA. The search for predictive imaging or biological/biochemical biomarkers for structural and symptomatic OA risk, onset, progression and response to therapy remains paramount [99], and more slowly progressive preclinical models could play a critical role in this. Longitudinal assessment of biomarkers in serum, urine, or synovial fluid can further contribute to unraveling correlations between changes in imaging and biochemical biomarkers with disease stage (time after onset) and structural pathology/histopathology. Much of the biomarker work focuses on specific matrix degradation products that are also commonly used in clinical trials [100]. Recently some interesting studies have attempted to cast a broader net for predictive markers, for instance through white blood cell microarrays in a horse model [101] and metabolic profiling of serum [102] or synovial fluid in sheep [103].

For patients and clinicians, clearly the outcomes that matter are pain relief and preservation of joint function. In large animal models, for instance in dogs and horses, it has been a longstanding practice to assess joint function, mainly through evaluating gait. Increasingly in small animal models, methods to assess disease sequelae of OA, including pain, sensitization, locomotion, gait, and even anxiety or depression, are incorporated as outcomes [104]. For instance, in a cruciate ligament transection model in mice, significant correlation was found between cartilage structural damage and motor function, gait, and thermal hyperalgesia [105]. These behavioral outcomes have yet to be standardized in the context of OA but it can be anticipated that comprehensive longitudinal assessment of structural disease, imaging and/or biochemical markers and pain, sensitization, and/or functional outcomes - as exemplified in recent clinical [106] and preclinical [107] studies - will increase our appreciation of the correlation between specific aspects of OA (and this may be different for different models mimicking distinct OA phenotypes).

## Is the model useful for studying prophylactic strategies or for studying structural arrest or reversal?

Many induced models of OA, particularly in rodents, are relatively rapidly progressive; thus, there may be a limited window for differentiating prophylactic from therapeutic effects. Progression of OA is generally slower in large animals, providing scope for evaluating timing of intervention, but they require more drug and are more expensive. The same OA induction method may have a very different time course and severity of disease in different species - for example, anterior cruciate ligament transection in sheep (mild, slow progression), goats (moderate, slow progression), dogs (moderate to severe, slow progression with phases of stability), and mice (severe, very rapid progression) (reviewed in [108]). Within a given species the severity and progression of OA will vary with the OA model, as demonstrated with different surgical induction methods in the mouse knee [109]. Interpretation of the prophylactic or therapeutic efficacy of an intervention, and how the findings translate to humans, requires in-depth understanding of the preclinical model used.

Spontaneous OA develops not only with advanced ageing (for instance, in male C57BL/6 mice) but also precociously (that is, during maturation rather than ageing) in a number of animals (for example, male Hartley guinea pigs and male STR/Ort mice). These spontaneous models are slowly progressive and amenable to evaluation of therapies for both prevention and structural arrest (for examples in guinea pig, see [110–112], and in STR/Ort mice, see [43, 113]). However, the variable onset and progression compared with induced models of OA may necessitate greater animal numbers to sufficiently power studies to detect therapeutic effect, and longer treatment times for prophylactic studies and thus more test compound and cost. It is also important to recognize that the underlying mechanisms that drive the onset and progression of spontaneous OA in these animals are not well defined, and may reflect specific subtypes of idiopathic human OA - for example, early SCB remodeling, meniscal ossification and ligament changes in Hartley guinea pigs [114–117] and systemic inflammatory/immune response in STR/Ort mice [118, 119].

Most preclinical OA studies test the efficacy of a given intervention on structural protection and usually in ‘prophylactic protocols’ - that is, treatment starts at the time or immediately after disease is induced. The direct translational relevance of this approach into the clinical situation is questionable, since even ‘early OA’ has structural changes in the joint at the time of diagnosis [98]. A prophylactic approach may be valid as an initial preclinical therapeutic trial given the current interest in specifically targeting post-traumatic OA [22], and it may provide the best opportunity to detect a therapeutic effect of the test compound or class of compounds - that is, proof of principle. However, when considering a broader patient population including those with established OA, testing interventions at different stages of disease in preclinical models is critical, as exemplified by the bisphosphonate [69, 70, 92] and osteoprotegerin [93] studies in rat OA models, where efficacy was quite different depending on when therapy was initiated. Intra-articular mesenchymal stem cells were found to be effective only when administered early in the mouse collagenase-induced arthritis model [73, 120]. Pharmacological augmentation of Runx1 on the other hand was effective in both prophylactic and therapeutic protocols in a surgically induced OA model in mice [121]. Clearly, to appreciate the therapeutic potential of any new agent, it will be necessary to test both prophylactic and therapeutic protocols across different models.

## Can the model be used to assess long-term toxicity?

**Fig. 2:**
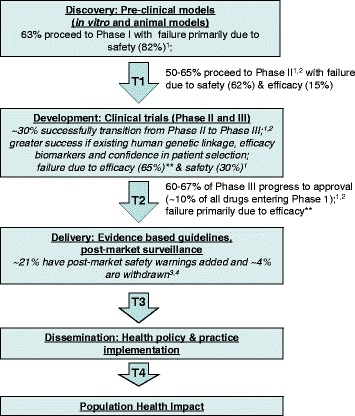
Drug development pipeline showing the translational phases (T1 to T4) from basic science discovery through to measurement of impact on population health. Failure of drug development programs can occur at all stages, but the major reasons differ with translational phase. **Efficacy failure is primarily due to poor biological rationale for the clinical trial: target linkage to disease not established or no validated models available (40 %) and indication selected does not fit the strongest preclinical evidence (20 %) [8]. ^1^[8], ^2^[142], ^3^[143], ^4^[144]

Since the chronic course of OA will require prolonged therapy aimed at improving quality of life, drug safety is a particularly important issue [99]. The single most common reason for closure of drug development programs is safety, usually during discovery phase mandatory toxicity testing [8] (Fig. 2). Toxicology studies are usually performed in healthy animals rather than in concert with the preclinical disease model, and may not involve use of animals with typical risks and co-morbidities associated with OA (such as age or obesity). It could be proposed that for a chronic pathology like OA, it may be advisable to test long-term blockade of a specific target in a disease-specific chronic model. With rapidly progressive disease models, such as rat MIA, often only evaluated for 3 to 4 weeks, long-term side effects or toxicity of therapy are unlikely to become apparent. The experience with unexpected musculoskeletal side effects in patients on prolonged broad-spectrum matrix metalloproteinase inhibitors, however, suggests that even routine use of long-term preclinical OA models may not have been predictive. To induce the syndrome in rats required high doses administered by osmotic pumps and careful histopathological screening or specific functional testing [122, 123]. More recently, the occurrence of rapidly progressive OA in non-target joints in patients enrolled in a clinical trial of anti-nerve growth factor [124] highlights the need for disease- and drug-specific toxicity models. In this case concomitant administration of nonsteroidal anti-inflammatory drugs (NSAIDs) has been implicated in the risk for side effects. It seems very unlikely that a preclinical model would routinely involve multi-drug therapy and model target and non-target joint disease.

## Predicting efficacy in preclinical models: how well have we done and what can we improve?

Placebo-controlled DMOAD trials for knee OA published in peer-reviewed journals since 1999 are listed in Table 1, juxtaposing clinical trial outcomes and findings in preclinical studies. A cursory evaluation of this table leads to the simple conclusion that preclinical models were poor predictors of clinical trial outcomes. However, when interpreted in light of the above discussion, it emerges that animal study design and use has been largely suboptimal. Preclinical testing was typically performed by treating prophylactically or early in induced models (mostly post-traumatic OA) in young and normal-weight animals, whereas clinical trials mostly focus on age/obesity-associated, established/late-stage (Kellgren-Lawrence grade 2 to 3) OA. Thus, the OA target population and preclinical phenotype are mismatched. Further, most preclinical studies reported are restricted to limited time points in one study in one animal model, in one species, and in one laboratory - that is, there is no testing for reproducibility. The animal studies usually evaluate a limited set of outcome parameters, and these parameters typically interrogate the mode of action of the drug more than assessing the overall joint health and animal well-being (that is, ‘exploratory’ rather than ‘confirmatory’ studies [125]). This limits translational value to the clinical DMOAD trial, where the primary outcome is joint space narrowing and there is usually a secondary symptomatic outcome.

Evidence for the predictive validity of OA animal models for testing efficacy of novel analgesics under development for OA pain is extremely limited. Recent clinical trials with antibodies against nerve growth factor showed clinical benefit, with reduction in joint pain and improved function [124], but results of preclinical testing of anti-nerve growth factor in OA models are not yet available in the public domain. Duloxetine, recently approved for the treatment of chronic musculoskeletal pain, was efficacious in a 13-week, randomized, double-blind, placebo-controlled trial in patients with symptomatic knee OA [126]. It has been tested in rat MIA, where it had a moderate effect on hind limb grip force deficit [127], and corrected weight-bearing deficit but not gait imbalance [128]. In the same model, local administration of a fatty acid amide hydrolase-1 inhibitor, which modulates endocannabinoids, significantly reduced hindlimb incapacitance [129], but a randomized, placebo-controlled clinical trial with an irreversible fatty acid amide hydrolase-1 inhibitor failed to induce effective analgesia in patients with painful knee OA [130]. The potential reasons for the different predictive utility of the model for patient outcomes are manifold. A recent, first of its kind, meta-analysis of preclinical studies of the effects of existing analgesic drugs (NSAIDs and opioids) in models of OA pain provides an analysis of factors that may impact the magnitude of the analgesic treatment effect in animal models of OA pain [131]. Improved understanding of how the analgesic effects of existing and novel compounds observed in various OA animal models relate to pain and disability in patients will be paramount to advance translation of symptom-modifying therapies.

## Conclusion

Design and use of animal models of OA can be deemed suboptimal, mainly with respect to OA phenotype, risk factors, and endpoints under evaluation. Suggestions for improvements that will enable translation in OA research are similar in other diseases [132] and include:

1. Better aligning of preclinical models and the clinical trial population - including OA disease phenotype, stage, age, sex and confounding co-morbidities.
2. Increasing the ‘translatability score’ of tested therapies [5, 6] by including evaluation in multiple OA models and species, and improving reproducibility (for example, replicating data in different laboratories or using ‘multi-institutional synchronous co-clinical trials in mice’ [132, 133]).
3. Standardizing preclinical outcome measures for both OA pathology and pain (for example, see collected papers in *Osteoarthritis and Cartilage* 2010, Supplement 3; and see [134]). This will not only enable comparisons between studies and research groups, but also permit evaluation of the relative efficacy of different interventions/treatments.
4. Making measured outcomes more clinically relevant by evaluating whole joint pathology (not just cartilage) and including measures of pain/disability in addition to structural pathology. Understanding how the outcomes measured in the animal model relate to the human disease may be improved through using clinically relevant imaging modalities in preclinical studies [135]. Changing emphasis from ‘statistically significant’ to ‘clinically significant’ effects is an issue pertinent to both preclinical and clinical research, requiring the establishment of ‘minimally clinically important differences’ (MCIDs) [136, 137]. The MCIDs for different outcomes in patients need to be translated to an equivalent ‘effect size’ in animal models, and then more universally applicable and standardized measures of efficacy in preclinical studies can be used, such as the ‘percentage responders’ (that is, that reach the MCID/effect size) and ‘number needed to treat’ [138].
5. Increasing the rigor of preclinical science and its reporting, including use of ARRIVE or other guidelines [139] enabling better use of meta-analysis of preclinical OA studies of structure and pain therapeutic modification [131, 140].
6. Embracing and making use of the One Health Initiative [141]. The concept behind this worldwide strategy is that interdisciplinary collaboration and communication between medical and veterinary health care and health research will provide multi-directional flow of knowledge and synergistic gains for both disciplines. Thus, beyond the use of induced OA models in laboratory animals to investigate pathophysiology and treatment, studying OA onset, progression and management in clinical veterinary practice provides a ‘real-world laboratory’ of naturally occurring disease cohorts with similar co-morbidities to humans (for example, obesity and ageing in dogs or exercise-induced trauma in athletic horses) for robust trials of newly developed therapies. This offers the potential to both benefit animals with OA and to inform human clinical trials and patient care.

Rather than concluding that preclinical models are not useful in translational medical research [4], this review has highlighted a number of issues that could be addressed to improve the predictive utility of OA animal models. Increasingly, researchers are incorporating these considerations into the design and reporting of studies in OA models. As both clinical and preclinical researchers improve comprehensive longitudinal assessment of structural disease, imaging and/or biochemical markers and pain, sensitization, and/or functional outcomes, our appreciation of OA phenotypes and their appropriate modeling will increase. It can be expected that this will result in better predictivity of preclinical findings for human translation and reduce failures.

## Abbreviations

DMM: Destabilization of the medial meniscus
DMOAD: Disease-modifying osteoarthritis drug
FDA: Food and drug administration
MCID: Minimally clinically important difference
MIA: Mono-iodoacetate
MRI: Magnetic resonance imaging
NSAID: Nonsteroidal anti-inflammatory drug
OA: Osteoarthritis
OARSI: Osteoarthritis research society international
SCB: Subchondral bone
